# Editorial: Enhancing psychological resilience and therapeutic adherence in organ transplantation

**DOI:** 10.3389/fpsyg.2026.1863413

**Published:** 2026-05-15

**Authors:** Victor Fernandez-Alonso, Maria Luisa Pistorio, Concetta De Pasquale, Semra Bulbuloglu

**Affiliations:** 1 Department of Nursing, Escuela Universitaria de Enfermería Cruz Roja-Universidad Autónoma de Madrid, Madrid, Spain; 2 Instituto de Investigacion Sanitaria Gregorio Maranon, Madrid, Spain; 3 Universita degli Studi di Catania, Catania, Italy; 4 Division of Surgical Nursing, Department of Nursing, Faculty of Health Sciences, Istanbul Aydın University, Istanbul, Türkiye; 5 Department of Midwifery, Faculty of Health Sciences, Istanbul Aydın University, Istanbul, Türkiye

**Keywords:** medication adherence, organ transplantation, patient-centered care, psychological resilience, psychosocial screening, quality of life

According to the WHO-ONT Global Observatory on Donation and Transplantation (GODT), more than 173,000 solid organ transplants were performed worldwide in 2024. Despite record activity, the GODT underscores persistent global inequities—transplantation still covers only a fraction of the worldwide need, with long waiting lists particularly in low- and middle-income settings [Martin et al., 2026; Global Observatory on Donation and Transplantation (GODT), 2026]. By the end of 2024, 94,644 patients remained on waiting lists and 8,591 people died while waiting. Donation after circulatory determination of death consolidated its role, and estimates a 2% global increase in 2024 (EDQM ONT, 2025). Organ transplantation is a highly effective therapy for end-stage organ failure, yet optimal outcomes depend on more than surgical technique and immunosuppression. This Research Topic, *Enhancing psychological resilience and therapeutic adherence in organ transplantation*, brings contributions that address psychosocial assessment, adherence risk, lived experience during critical illness, and scalable supportive interventions.

A central message from this Topic is that adherence vulnerabilities can be present before transplantation and may be associated with modifiable psychological factors. Incorporate iterative psychosocial and adherence assessment into routine pre-transplant evaluation and waitlist management, with escalation pathways for uncertainty, affective symptoms, and observed non-adherence patterns. Sanborn et al., described in thoracic transplant candidates, high rates of pre-transplant non-adherence are reported, with links to affective variables and uncertainty, supporting the clinical value of early, structured adherence assessment and follow-up during the waitlist period. Building on the “why screen early” rationale, a complementary contribution addresses the “how” by developing and evaluating transplant-specific problem lists designed to identify psychosocial distress domains relevant both before and after transplant. Müller et al., reported such tools can support standardized clinical workflows—e.g., systematic detection of concerns in daily functioning and social participation—with clearer pathways to targeted psychosocial support. Importantly, emotional status in transplant candidates is not static. Pennington et al., in the longitudinal multicenter data in heart transplant candidates demonstrate dynamic trajectories of emotional affect prior to transplant, implying that single time-point screening may miss clinically relevant changes and that repeated assessment may be more informative for risk stratification and timely intervention.

ICU and perioperative pathways should include structured communication practices, anticipatory guidance for sensory and cognitive stressors, and access to psychosocial support proportionate to illness severity and patient vulnerability. The perioperative period—particularly ICU care—emerges as a phase in which the patient experience can be dominated by sensory overload, communication barriers, and disorientation. A qualitative phenomenological study of heart and lung recipients, by Ugenti et al., in intensive care characterizes these “lived experience” elements and highlights the clinical value of empathic care and effective communication as modifiable factors that may reduce distress and improve perceived support. In parallel, qualitative work in liver transplant recipients receiving perioperative ECMO reports benefit-finding and perceived mastery under critical circumstances, particularly when psychological support is present. These findings of Lei et al., support the feasibility of addressing resilience processes even during complex perioperative courses, rather than deferring psychosocial care exclusively to post-discharge phases.

Post-transplant care should combine (i) evidence-informed psychological interventions, (ii) patient-centered adjuncts such as peer mentoring where feasible, and (iii) ongoing monitoring aligned with changing emotional and adherence-related risk. Several contributions emphasize that transplantation can represent a meaningful shift in perceived health and psychological functioning. Comparative analysis across chronic kidney disease modalities, by Pignataro et al. suggests differences in psychological wellbeing between conservative therapy, dialysis, and kidney transplantation, with transplantation frequently associated with improved wellbeing and the potential identification of biobehavioral correlates (e.g., exploratory markers linked to wellbeing). Beyond general wellbeing, the Topic highlights scalable support models. A qualitative study, by Kumnig et al., describing a patient-centered peer mentoring program for kidney transplant recipients underscores the distinctive value of lived-experience support—normalization of challenges, practical problem-solving, and social connection—delivered alongside standard multidisciplinary follow-up. At the intervention level, a systematic review synthesizes evidence for psychological approaches used in transplant populations, including structured therapies such as CBT and mindfulness-based methods. While the review, by De Pasquale et al., supports potential effectiveness, it also identifies a need for standardized outcomes and longer follow-up, strengthening the case for harmonized evaluation frameworks and longitudinal designs.

Finally, this Topic extends beyond recipients to the broader social context that determines access to transplantation. A study in by Tomaino et al., shows that open group dialogue can shift attitudes toward post-mortem organ donation, illustrating the role of health psychology in public engagement and donation-supportive culture, and highlighting that psychosocial strategies can contribute at a systems level.

Three priorities follow directly from the evidence assembled here: (i) Standardize and repeat psychosocial assessment (including distress and adherence risk) across key phases—pre-transplant, perioperative/ICU, and post-transplant—recognizing that emotional status and adherence vulnerabilities change over time. (ii) Develop and implement scalable interventions that combine professional psychological care with patient-centered supports (e.g., peer mentoring) and robust care processes (e.g., structured communication in ICU settings). (iii) Strengthen the evidence base through longitudinal, multicenter research with harmonized outcomes, enabling meta-analysis and supporting translation into routine practice.

This Research Topic calls for a paradigm shift: to systematically integrate psychosocial screening, to combine professional and peer supports, and to study not only pathology but also the mechanisms of benefit-finding that turn a successful operation into a fulfilling life. We invite transplant teams to adopt these practices now, and the research community to generate longitudinal, standardized evidence that will accelerate their implementation. As Topic Editors, we hope this Research Topic assists transplant teams in operationalizing psychosocial screening, adherence-focused pathways, and resilience-supportive care to improve patient experience and outcomes across the transplant journey.
